# PowerPoint^®^ Presentation Flaws and Failures: A Psychological Analysis

**DOI:** 10.3389/fpsyg.2012.00230

**Published:** 2012-07-17

**Authors:** Stephen M. Kosslyn, Rogier A. Kievit, Alexandra G. Russell, Jennifer M. Shephard

**Affiliations:** ^1^Center for Advanced Study in the Behavioral Sciences, Stanford UniversityStanford, CA USA; ^2^Department of Psychology, University of AmsterdamAmsterdam, Netherlands; ^3^Department of Psychology, Stanford UniversityStanford, CA, USA; ^4^Division of Social Science, Harvard UniversityCambridge, MA, USA

**Keywords:** PowerPoint^®^, electronic slide show, presentation graphics, visual display design, clear communication, educational media, conveying information

## Abstract

Electronic slideshow presentations are often faulted anecdotally, but little empirical work has documented their faults. In Study 1 we found that eight psychological principles are often violated in PowerPoint^®^ slideshows, and are violated to similar extents across different fields – for example, academic research slideshows generally were no better or worse than business slideshows. In Study 2 we found that respondents reported having noticed, and having been annoyed by, specific problems in presentations arising from violations of particular psychological principles. Finally, in Study 3 we showed that observers are not highly accurate in recognizing when particular slides violated a specific psychological rule. Furthermore, even when they correctly identified the violation, they often could not explain the nature of the problem. In sum, the psychological foundations for effective slideshow presentation design are neither obvious nor necessarily intuitive, and presentation designers in all fields, from education to business to government, could benefit from explicit instruction in relevant aspects of psychology.

## Introduction

PowerPoint^®^, Keynote^®^, and their electronic slideshow siblings have become pervasive means of communication. Indeed, in 2001, Microsoft estimated that an average of 30 million PowerPoint^®^ presentations were given *each day* – and we can only imagine what that number is today (Parker, 2001). Presentations using such computer programs are commonplace in academia, business, government, the military, and even K-12 schools. PowerPoint^®^ has been used to teach subjects as varied as the three-dimensional structure of the larynx (Hu et al., 2009), prosthetics and orthotics (Wong et al., 2004), how to perform a testicular exam (Taylor et al., 2004), developmental psychology (Susskind, 2008), and physics (Gunel et al., 2006).

Observers have offered varied opinions about PowerPoint^®^ and the value of presentations that utilize it (e.g., for overviews, see Farkas, 2006; Stoner, 2007), and considerable empirical research has been conducted on the use of the medium (e.g., for reviews, see Susskind, 2005, 2008; Levasseur and Sawyer, 2006; Savoy et al., 2009). One set of this research has examined whether using this medium improves learning or comprehension. For example, Gunel et al. (2006) found that students learned more physics from PowerPoint^®^ presentations than from chapter summaries, whereas other researchers have reported that students remember about the same amount of material following PowerPoint^®^ presentations as they do following other media (such as overheads and use of the blackboard; e.g., Szabo and Hastings, 2000; Beets and Lobingier, 2001; Campbell et al., 2004; Apperson et al., 2006; Experiments 1 and 3; Susskind, 2005, 2008). Indeed, some studies find that PowerPoint^®^ actually impairs learning. For example, Savoy et al. (2009) showed that students recalled more verbal information from a traditional lecture than they did when that information was presented verbally during a PowerPoint^®^-based lecture.

Researchers have also examined whether students prefer PowerPoint^®^ presentations to other types of presentations. The results of such studies generally indicate that students do like PowerPoint^®^ presentations (Susskind, 2008; Savoy et al., 2009). Apperson et al. (2008) asked psychology students what in particular they liked about PowerPoint^®^ presentations, and found that they like to see lists built up one item at a time and that they also like outlines of key phrases, graphics, relevant sounds, and colored backgrounds. However, it is worth noting that Mahar et al. (2009) compared memory retention after an entire list had been presented simultaneously to retention when each item on the list had been presented individually, and found better memory (but only for two of the nine items on their test) in the simultaneous condition.

Even this brief overview of the literature reveals that the results are mixed. One reason for the varied results may lie in differences in the quality of the presentations: the medium is not the entire message; any medium can be used effectively or ineffectively. In our view, the key is not whether or not PowerPoint^®^ is used, but rather *how* it is used. Nobody should be surprised if a PowerPoint^®^ lecture utilizing poorly designed slides or presented by an inadequately prepared speaker is not effective. In this article, we use psychological principles to develop objective guidelines for slideshow design, slide preparation, and presentation execution, and use these guidelines to assess flaws in PowerPoint^®^ slides and presentations.

In the three studies reported here, we empirically extend ideas presented in Kosslyn (2007, 2010) and analyze how well PowerPoint^®^ slideshows, slides, and presentations respect principles of human perception, memory, and comprehension. Specifically, we hypothesize that the psychological principles presented in the following section are violated in PowerPoint^®^ slideshows across different fields (Study 1), that some types of presentation flaws will be noticeable and frustrating to audience members (Study 2), and that observers will have difficulties in identifying violations in the case of graphical displays in individual slides (Study 3).

## Eight Cognitive Communication Principles

Levasseur and Sawyer (2006) note in their extensive review that remarkably little research provides direct guidelines for designing presentations (see also Suchoff, 2006). However, much research on basic psychological processes has been conducted that can be used to formulate such guidelines. This observation has been appreciated by many researchers, who have understood the fundamental point that effective displays must play to the strengths of human information processing and must avoid relying on the weaknesses of such processing (e.g., Aspillaga, 1996; Helander et al., 1997; Mejdal et al., 2001; Vekiri, 2002; Watzman, 2003; Stanney et al., 2004).

We acknowledge that the following formulation is but one of several possible ways to organize these guidelines within the conventional view of human information processing, but this one (from Kosslyn, 2007) appears to capture the major points of agreement among researchers. Moreover, this set of principles is a convenient way to organize the specific rules we examined, and it is these rules that identify the specific aspects of psychological processing we investigated. Thus, in addition to grouping the data according to the principles, we provide the data for each individual rule – which also allows readers to consider the results for those rules of particular interest.

In formulating our eight Cognitive Communication Principles, we began with a task analysis; that is, we considered what the viewer must do to understand a presentation. This analysis rests on the now-standard view that perception and comprehension can be decomposed into distinct classes of information processing operations (e.g., see Reisberg, 2006; Smith and Kosslyn, 2006), which underlie *encoding*, *working memory*, and *accessing long-term memory*. We focus here primarily on the visual modality because the visual aspects of the slide design are a major factor of PowerPoint^®^ presentations (although the presenter’s speaking style and structuring of the presentation are also important, as we address in Study 2). In what follows, we first present the basic findings about information processing that led us to formulate a specific principle, and then explain the principle and how we operationalized it. Crucially, because all of these principles stem from known bottlenecks in information processing, slideshows, slides, and presentations that violate them will tax human information processing.

In what follows, although we organize the information processing operations into three categories (encoding, working memory, and accessing long-term memory), we do not mean to imply that these processes work in lockstep sequence. Rather, by focusing on the operations, we are led to define the bottlenecks that can disrupt presentations.

### Encoding processes

When viewing a sequence of slides, audience members first must encode what they see; if they fail to encode material, it may as well not exist. We formulated three principles based on essential facts about encoding.

#### Discriminability

The initial step of encoding requires noticing the to-be-encoded material, which requires patterns to be clearly different from the background and from other patterns. This leads us to formulate *the Principle of Discriminability*: two properties (such as two colors, degrees of gray, or sizes) cannot convey different information unless they differ by a large enough proportion to be easily distinguished (Woodson et al., 1992). This principle underlies camouflage, which occurs when two properties are so similar that they are not distinguishable – and there’s no place for camouflage in a presentation. In addition, we humans are not very good at discriminating among differences in the sizes of areas: we tend to underestimate areas, and do so to an increasingly large degree as the area increases (Stevens, 1975). The Principle of Discriminability has the following corollaries:

(1) Unless typefaces are large enough, letters cannot easily be distinguished from each other.
(2) If the color of text or graphics is not clearly distinct from the color of the background on which they appear, they cannot be readily distinguished.
(3) Typefaces in which letters are similar (because they are visually complex, all upper case, all italics, or all bold) cannot be easily read.
(4) Because patterns that are registered by separate “spatial frequency channels” are easily distinguished (Stromeyer and Klein, 1974; De Valois and De Valois, 1990), viewers can easily distinguish points that are about twice as thick as the lines that connect them and can easily distinguish texture patterns in which elements (e.g., stripes) vary by at least 2:1 (which will also avoid distracting “visual beats”).
(5) Similarly, because orientations that differ by at least 30° are processed by different “orientation channels” (Thomas and Gille, 1979; De Valois and De Valois, 1990), viewers can easily distinguish orientations of the lines in different regions when they vary by at least 30°.
(6) Finally, because of the way that different wavelengths of light focus on the retina, we have difficulty bringing cobalt blue (which is a mixture of red and blue) into focus, and we have difficulty focusing on red and blue at the same time (combinations of these colors tend to “shimmer”); thus text or other fine lines are difficult to distinguish if they are rendered in deep blues, and boundaries defined by adjacent red and blue regions are distracting.

#### Perceptual organization

Once material is noticed, processes that underlie figure/ground segregation organize the input into perceptual units that often correspond to objects (including words and graphics). This observation leads us to formulate the *Principle of Perceptual Organization*: people automatically organize elements into groups, which they then attend to and remember (Larkin and Simon, 1987; Gropper, 1991; Aspillaga, 1996; Helander et al., 1997; Vekiri, 2002). Such groups emerge in numerous ways (Palmer, 1992). Classical grouping “Laws” specify the perceptual conditions that lead viewers to see elements as a single group. For example, we tend to group together elements that are nearby (the so-called “Law of Proximity”). That is, we see “xxx xxx” as two groups of three each, not six separate x’s. Similarly, we tend to group together elements that appear similar (the so-called “Law of Similarity”). For instance, we see “///\\\” as two groups, not six separate lines.

Grouping also can be imposed by lines, such as those used in complex tables to define specific regions, which makes the tables easier to read. In addition, some visual dimensions are automatically grouped; these dimensions are “integral,” and cannot easily be considered independently. For example, height and width are not easily seen as distinct, but rather are organized into a single shape – and viewers register the overall area, not the two dimensions separately (Macmillan and Ornstein, 1998). Other integral dimensions are hue, lightness, and saturation, which cannot easily be attended to independently; rather, our visual systems tend to group them into a single color – and thus these different dimensions of color cannot easily be used to convey separate measurements. The Principle of Perceptual Organization has the following corollaries:

(1) Labels initially are seen as applying to the closest graphic element.
(2) Common color organizes parts of a display into a group, even if they are separated in space.
(3) Common patterns or orderings organize parts of a display into a group, even if they are separated in space. For example, when the elements in a key (e.g., small squares of different colors, each associated with a label) are ordered the same way as the corresponding parts of the display (e.g., bars in a bar graph), the elements in the key will be grouped appropriately with the corresponding parts of the display.
(4) Because shape and location largely are processed separately in the visual system (Ungerleider and Mishkin, 1982), an element in one location will not always be easily grouped with elements in other locations. Hence, it is useful to construct a display so that separated-but-related elements are explicitly grouped (e.g., by using inner grid lines to group the tops of bars in a bar graph with locations along the *Y* axis at the left of the graph, if specific point values are to be read).
(5) Comprehension is impaired by spurious groups, which form accidentally because of the grouping laws (e.g., such as those that can form when pairs of measurements for two or more categories are plotted in the same scatter plot, or when a banner at the top of a slide groups with specific objects in the display because of proximity or similarity).

#### Salience

Not all perceptual units can be processed simultaneously, if only because they are in different locations and the visual acuity of the eye is greatest at the fovea (and hence whatever people fixate their gazes on will be encoded with higher resolution). Thus, some units are encoded in detail before others. Attention selects which patterns will be processed in detail, and attention is automatically drawn to what is different; we immediately notice the nail that sticks above the floorboards or the one red light in a group of green lights. The *Principle of Salience* states: attention is drawn to large perceptible differences. In fact, a part of the brain (the superior colliculus) operates as an attentional reflex, automatically drawing our visual attention to large differences among stimuli (Posner, 1980; Glimcher and Sparks, 1992; Krauzlis et al., 2004). The Principle of Salience has the following corollaries:

(1) Because movement is a particularly salient cue (Buchel et al., 1998), animation captures and directs attention.
(2) Text with a distinct format (color, size, or typeface) draws attention.
(3) Visual disparities draw attention. For example, we notice a wedge exploded from a pie chart because the boundary has been disrupted; however, if more than one or two wedges are exploded, the boundary becomes so disrupted that the separated pieces are no longer salient.
(4) If salience is aligned with importance (Helander et al., 1997), the more important aspects of the slide (e.g., the title or topic sentence) or of an illustration (graph, diagram, demo) draw the audience members’ attention – which will also enhance later memory for those aspects (Schmidt, 1991).
(5) A special case of salience arises with colors: because of a quirk in how the corneas diffuse light and project it onto the retinas, “warm” colors (i.e., those with relatively long wavelengths, such as red) seem closer to the viewer than do “cool” colors (i.e., those with relatively short wavelengths, such as blue; see Held, 1980; Allen and Rubin, 1981; Travis, 1991). Thus, if warmer colors are used for lines that pass beneath other lines, viewers will experience an annoying illusion in which material at the back appears to be struggling to move forward.

### Working memory

After visual patterns are encoded, they must be integrated. In a typical presentation, material must be integrated in working memory over the course of multiple slides, each of which may require multiple eye fixations. This integrating process is a prelude to fully comprehending both individual displays and the entire presentation. The high demands on working memory lead to the following two specific principles.

#### Limited capacity

One of the key facts about working memory is that it has a very limited capacity (e.g., Baddeley, 2007). The *Principle of Limited Capacity* states that people have a limited capacity to retain and to process information and will not understand a message if too much information must be retained or processed (Smith and Mosier, 1986; Shneiderman, 1992; Helander et al., 1997; Lund, 1997; Sweller et al., 1998). The Principle of Limited Capacity has the following corollaries:

(1) The amount of information that people can retain in working memory is defined in terms of psychological units, such as the perceptual groups produced by the classical perceptual grouping laws. We can hold in working memory only about four such units (Cowan, 2001). However, each of these units in turn can comprise four units – and thus hierarchical organization can enhance our ability to hold information in mind.
(2) In general, humans can track the movement of only about four units at the same time (Intriligator and Cavanagh, 2001 – although under special circumstances, more can be tracked; Alvarez and Franconeri, 2007).
(3) Eliminating the need to search for labels – for instance by directly labeling items in a display rather than using a key – reduces processing load (c.f. Sweller, 1999).
(4) Audience members need time to process the information that is presented.
(5) However, conversely, if slides fade-in or fade-out very slowly, the audience members may organize them incorrectly and then have to break their initial organization – which also requires effort (Potter, 1966).

#### Informative change

Because working memory has limited capacity, extraneous information can easily overwhelm it at the expense of relevant information. But, by the same token, visual or auditory cues can be used to help organize the information. According to the *Principle of Informative Change*, people expect changes in perceptual properties to carry information, and expect every necessary piece of information to be conveyed by such a perceptible change. Indeed, the very concept of “information” has been defined in terms of change: only when there is a change is information conveyed (Shannon, 1948). The Principle of Informative Change has the following corollaries:

(1) Audience members assume that words, graphics, or other changes in appearance convey new information (Smith and Mosier, 1986; Shneiderman, 1992; Aspillaga, 1996; Helander et al., 1997; Lund, 1997). For example, audience members assume that new information is being conveyed by changes in the appearance of the background, bullet points, or color or typeface of text. Random or arbitrary changes in appearance, in transitions between slides, or in terminology (“fowl” versus “bird”) can lead the audience astray.
(2) Clearly marking the beginnings and ends of sections of a presentation (for instance by presenting a title or concluding slide with a distinct format, typeface, or background) helps audience members follow the presentation.

### Accessing long-term memory

Our task analysis leads us to posit a third class of relevant processes. Encoding information and integrating it appropriately would be useless if the meaning of the material were not extracted. In order to ascribe meaning to stimuli, one must compare them with material previously stored in long-term memory; it is only by retrieving associated information that we comprehend the import of what we see. The following principles address factors that affect the ease of accessing long-term memory and activating the relevant stored information.

#### Appropriate knowledge

Meaning can be ascribed to a pattern only if the person has the requisite information already stored in long-term memory; to reach an audience, the presenter must make contact with what the audience members already know (Osman and Hannafin, 1992; Shneiderman, 1992; Fleming and Levie, 1993; Lund, 1997; Schwartz et al., 1998). The *Principle of Appropriate Knowledge* states: communication requires prior knowledge of relevant concepts, jargon, and symbols. If the presenter relies on novel concepts, jargon, or symbols, the audience members will fail to understand. The Principle of Appropriate Knowledge has the following corollaries:

(1) Unfamiliar (for that audience) concepts, conventions, formats, terminology, and symbols may not be understood. Moreover, when they are understood, they will likely require effortful processing (which will be accomplished only if the audience is highly motivated).
(2) If unfamiliar (for that audience) concepts, conventions, formats, terminology, and symbols are absolutely necessary to convey the message, they must be explicitly introduced and explained – and such explanations are more effective if they draw on information that is familiar to the audience.

#### Compatibility

The meaning of a stimulus will be difficult to extract if the interpretation of its surface properties (such as the size or color) is inconsistent with its symbolic meaning. The *Principle of Compatibility* states that a message is easiest to understand if its form is compatible with its meaning (Vessey, 1991; Woodson et al., 1992; Vekiri, 2002; Speier, 2006). This principle is perhaps most evident when it is breached, as demonstrated by the classic “Stroop effect” (MacLeod, 1991). In the Stroop effect, people have more difficulty naming the color of the ink used to print words when the words name a different color from the ink (e.g., blue ink used to print the word “red”) than when words name the same color (e.g., blue ink used to print the word “blue”). We register both the surface properties (e.g., the color of the ink) and the meaning. This principle applies across perceptual modalities, and thus comprehension generally is best when audio and visual contents coordinate with text and the overall message that is being conveyed; inappropriate sounds and visuals will interfere with comprehension. The Principle of Compatibility has the following corollaries:

(1) Because hue is a metathetic (values on metathetic dimensions are arranged qualitatively) variable, variations in hue are not seen automatically as signaling different amounts of a quantity; in contrast, because saturation and intensity (or brightness) are prothetic variables (values on prothetic dimensions are arranged quantitatively), variations along these dimensions do line up with variations in quantities (Stevens, 1975).
(2) Animation interferes with comprehension if it does not fit the natural movements of the object (e.g., a picture of a car should not drop down from the top), and sounds and slide backgrounds will interfere if they are not appropriate for the topic (a floral background, or the sounds of birds chirping, are not compatible with a presentation about carbon reservoirs in the ocean).
(3) Viewers most easily interpret icons that depict the typical examples of represented items. For example, a picture of a duck effectively illustrates “water fowl” but not “pet bird,” and vice versa for a picture of a canary (Rosch et al., 1976).
(4) Old-fashioned looking typeface would send a conflicting message if used in a written description of a high-tech device.
(5) Because they make different sorts of information explicit, different sorts of graphics are appropriate for making different points (Tversky et al., 2002). Line graphs (rather than bar or mixed graphs) illustrate trends effectively because the continuous variation in the height of a line in a graph directly indicates the continuous variations of a measurement. Similarly, crossing lines in a graph indicate interactions more effectively than sets of bars. Bar graphs illustrate specific values (not trends) effectively because the discrete heights of the bars directly indicate specific measurements. Maps illustrate complex information about geographic territories or show alternate routes to a destination. Charts effectively illustrate organizational structure, a sequence of steps, or processes over time (“flow charts”).

#### Relevance

Finally, the message must be calibrated so that neither too much nor too little information is presented for a specific audience (Grice, 1975). (Note: the Principle of Informative Change states that, given a specific message, the requisite information must be provided – the present point is about providing an appropriate message in the first place.) It is clear that not providing enough information is a problem, but perhaps it is less immediately evident that providing too much information (such as extraneous graphics, text, or audio) is also a problem. Presenting too much information is a problem in part because this forces viewers to search for the relevant information, which requires effort (e.g., Wolfe, 1998). The *Principle of Relevance* states that communication is most effective when neither too much nor too little information is presented (Smith and Mosier, 1986; Woodson et al., 1992; Vekiri, 2002; Bartsch and Cobern, 2003). This principle has the following corollaries:

(1) To decide what is too much or too little, one must know about the nature of the message: depending on what the intended point is, specific information can be necessary or extraneous.
(2) When attempting to understand information, people (largely unconsciously) organize it into a narrative (c.f. Wagoner, 2008; Karns et al., 2009). Defining the topic and presenting a roadmap at the outset (in an outline or other overview) facilitates this process.
(3) Graphics (photos, drawings, graphs, diagrams), audio, and video can provide detail to illustrate the relevant concepts clearly. However, pictures are often ambiguous (Wittgenstein, 1953/2001), and when they are ambiguous labels can clarify them.

In the following study we use these Cognitive Communication Principles, and the specific rules that grow out of them, to evaluate a wide variety of slideshows and slides. We ask the following questions: first, are violations of the principles common? Given that many corollaries of these principles are not intuitively obvious, we hypothesize that we will find many violations. Second, are the violations equally common in different fields? Because human beings are preparing the slides in all cases, we have no grounds for hypothesizing differences in the frequency of violations in the different fields.

## Study 1

Levasseur and Sawyer (2006) noted that there is remarkably little research on how slides should be designed so that they function effectively. One possible reason for this dearth is that many may feel that “good design” is intuitively clear to most people, and hence there is no reason to study it. Consistent with this conjecture, some have claimed that the sorts of psychological principles just discussed are obvious, and rarely if ever would be violated in a presentation. To evaluate this supposition, we examined a sample of PowerPoint^®^ slideshows. Specifically, we used a stratified sampling procedure to examine a selection of slideshows in five categories: Research (academic), Education, Government, Business, and Miscellaneous. We formulated 137 ways (each specified in a rule) in which the eight principles just summarized could be violated (see Table 1).

**Table 1:** **List of rules for each principle and the proportion of presentations violating each rule (according to strict and liberal criteria) for Study 1**.

	Violations (Strict)	Violations (Liberal)
**APPROPRIATE KNOWLEDGE**		
Unusual bullet symbols are used	0.379	0.257
Non-standard or unfamiliar display formats are used	0.343	0.300
The key is not at the top right of a single panel or centered over multiple panels	0.279	0.264
The title is not at the top of the slide	0.057	0.057
Symbols are potentially ambiguous for the audience	0.057	0.050
Standard conventions for fonts are not used	0.014	0.014
Terms do not convey the appropriate denotations and connotations	0.014	0.014
**COMPATIBILITY**		
The layout of a chart is not compatible with the subject matter	0.107	0.079
A line graph is not used to display trends	0.093	0.093
A line graph is not used when the *X* axis uses a continuous scale	0.086	0.086
Font is incompatible with its connotations (sans serif implies modern, technological; serif implies traditional)	0.064	0.036
The background pattern is inappropriate to the main point of the display	0.057	0.050
Sounds are not appropriate for the topic and point being made	0.021	0.021
More inclusive categories are not higher in an organizational chart	0.021	0.021
The style of photos or clipart is not compatible with the message	0.014	0.007
Sounds, text, and graphics are not coordinated	0.014	0.014
A bar graph is not used to illustrate differences between specific point values	0.014	0.014
Animations/videos are not compatible with the represented object or event	0.007	0.007
Saturation and lightness are not varied for hues that indicate greater amounts (which is a problem because variations in hue alone are not naturally seen as corresponding to variations in amount)	0.007	0.007
A line graph is not used to display interactions over two levels on the *X* axis	0.007	0.007
A bar graph is not used when more than two values are on an *X* axis that does not show a continuous scale	0.007	0.007
A chart is not used to illustrate sequences of steps over time	0.000	0.000
A map is not presented when more than one route is possible	0.000	0.000
Mixed bar/line displays are used to show interactions	0.000	0.000
Colors are incompatible (given common conventions) with the meaning of the colored elements	0.000	0.000
**DISCRIMINABILITY**		
All uppercase, all italics, or all bold typefaces are used	0.807	0.557
Words are not large enough (i.e., greater than 20 point) to be easily read	0.664	0.650
Deep, heavily saturated blue is used for text or graphics	0.550	0.429
Entries in a table are too small to be read easily	0.514	0.486
Underlining is used	0.464	0.257
Colors shimmer	0.421	0.393
Photos and clipart become too grainy when inserted into the slide	0.414	0.314
Red and blue are used in adjacent regions	0.357	0.236
Information-conveying visual properties are not discriminable	0.129	0.129
Text cannot be easily discriminated from the background	0.129	0.129
Hues are not well separated in the spectrum	0.114	0.114
Different lines connect different points, but the lines are not easily discriminated	0.064	0.064
The foreground and background are not easily discriminable	0.057	0.050
Labels and patches in a key are difficult to tell apart	0.057	0.050
Points or symbols connected by different lines are not easily discriminated	0.043	0.043
If lines connect discrete points, the points are not at least twice as thick as the line	0.043	0.043
Adjacent colors have similar lightness	0.036	0.029
Visual beats occur	0.029	0.014
Visually complex fonts are used	0.021	0.014
Double-spaced bullets are used	0.014	0.014
Dashes in lines do not differ by at least 2–1	0.014	0.007
Sounds are not high fidelity	0.000	0.000
Orientation of textures used to fill patterns does not vary by at least 30° of arc	0.000	0.000
Spacing of texture patterns with similar orientations does not vary by a ratio of at least 2–1	0.000	0.000
Area of elements is used to convey precise quantities	0.000	0.000
**INFORMATIVE CHANGES**		
Visual or auditory characteristics change even when they do not signal a change in information	0.721	0.564
There is no crisp ending to signal that the presentation, or a given part, is over	0.643	0.593
Serif and sans serif are mixed arbitrarily	0.371	0.207
A consistent and distinctively formatted slide does not signal the beginning of each new part/group of the presentation	0.314	0.300
Different bullet symbols are used for entries in a list of similar items	0.257	0.186
Different transitions are used randomly for different slides	0.057	0.057
The same terminology is not used in labels and surrounding text	0.000	0.000
Tonal quality or volume is varied randomly	0.000	0.000
**LIMITED CAPACITY**		
Bulleted items are not presented individually, growing the list from the top to the bottom	0.957	0.943
More than two lines are used per bulleted sentence	0.914	0.871
More than four bulleted items appear in a single list	0.914	0.879
Hierarchical organization of lists is not used, with no more than four items at each level	0.857	0.836
Slides contain more than what can be read aloud in about 1 min	0.314	0.314
Complex displays are not built up a part at a time	0.271	0.264
Viewers are expected to read a complex table	0.186	0.164
More than four perceptual units are presented in one panel of a graphic	0.164	0.164
The conceptual structure or outline is not organized hierarchically into groups of no more than four elements	0.093	0.093
Slides fade-in or fade-out too slowly	0.057	0.036
Content elements are not labeled directly whenever space permits	0.021	0.021
A key is used when direct labels could be used instead	0.014	0.014
More than four separate perceptual groups are moved simultaneously	0.007	0.007
Hierarchical labeling of graphics is not used	0.007	0.007
Multiple pie graphs are used to compare corresponding parts even though the proportions vary greatly	0.007	0.007
**PERCEPTUAL ORGANIZATION**		
In tables with more than two rows and two columns, grid lines are not included	0.150	0.150
The title is too close to other words or patterns and groups with them	0.100	0.086
Parts of background patterns group with parts of the foreground	0.079	0.079
In a key, labels and patches fail to group together	0.079	0.071
The space between bar clusters is less than the width of two bars	0.064	0.057
Patches in keys and their corresponding content elements are in different orders	0.064	0.050
Labels are not grouped with the appropriate elements of the display	0.057	0.057
A banner at the top is not clearly distinct from the other material	0.007	0.007
An inner grid is not used in a graph when precise values are important	0.007	0.000
Corresponding bars are not arranged in the same way	0.007	0.007
Words in the same label are not close together and typographically similar	0.000	0.000
Hue, lightness, and saturation specify different measurements (which is a problem because we cannot easily see these properties as independent)	0.000	0.000
Portions of the same text, line, or graphic move separately	0.000	0.000
Height and width are used to specify separate variables	0.000	0.000
There is no space between bar clusters	0.000	0.000
Corresponding bars are not marked in the same way	0.000	0.000
**RELEVANCE**		
Bullets do not introduce topic sentences/phrases or specific cases	0.471	0.471
Either more or less detail than required for the point is presented	0.379	0.379
*X* and *Y* axes are not clearly identifiable and appropriately labeled	0.207	0.207
Problem, question, or topic of the presentation is not defined	0.150	0.121
Tables show more than the information needed to make the point	0.136	0.136
Gratuitous animation, which obscures rather than illuminates the point, is presented	0.121	0.079
Every graphic or table, as well as each component of the content material, is not labeled (unless the identity is self-evident)	0.086	0.079
Gratuitous graphics, videos, or sounds are presented	0.071	0.064
Photos or clipart are named with a word or phrase that does not bear directly on the point	0.057	0.050
Photos and clipart do not: define the context, introduce an abstract idea, or evoke a specific emotion	0.050	0.036
An overview of a list is not presented	0.029	0.029
Complex concepts are not illustrated clearly with graphics (displays, videos, sounds, or animations)	0.029	0.029
If sounds are presented, they do not provide useful and pertinent information	0.021	0.021
Sounds are used without explanation or labeling	0.021	0.021
Bars extend beyond the end of the scale	0.014	0.007
A table is not presented when needed (i.e., when specific values are important)	0.007	0.007
Labeled routes in a map are not important, or important routes are not labeled	0.000	0.000
Labels are not provided when precise amounts are relevant	0.000	0.000
Important distances are not labeled directly	0.000	0.000
**SALIENCE**		
Different colors are not being used for emphasis or to specify	0.329	0.314
The most important content element is not the most salient	0.321	0.314
The color of the text is more salient than the color of the title	0.286	0.243
Color makes less important elements salient	0.243	0.236
The salience of lines or bars does not reflect relative importance	0.136	0.129
The background pattern is very salient	0.100	0.093
The title is not typographically distinct	0.093	0.093
Illustrations do not face the center of the slide (and hence direct attention to the side)	0.064	0.064
Warm colors do not define the foreground	0.043	0.043
More salient labels are not used to label more important components of the display	0.029	0.029
More than 25% of the wedges in a pie are exploded	0.029	0.029
Sounds do not grab viewers’ attention appropriately	0.021	0.021
**“OVER-DETERMINED”**		
The title of a very complex slide is not presented before presenting the content elements	0.171	0.164
The title of a slide does not focus attention on the most important point	0.093	0.071
Irrelevant words or graphics are easily distinguishable from the background	0.093	0.079
Different types of data are graphed in a single display even when they are unrelated	0.071	0.064
Pictures and icons used as labels do not evoke the appropriate concepts	0.050	0.050
Wedges in a pie graph are not arranged in a simple progression	0.043	0.043
Shapes of meaningful regions are not easily identifiable	0.036	0.036
The same size and font is not used for labels of corresponding components	0.029	0.021
A map is not used to label complex sets of information about a territory	0.021	0.021
A chart is not used to convey overall organizational structure	0.014	0.014
Viewers must read moving words	0.014	0.014
Multiple panels are not being used to highlight specific comparisons	0.007	0.007
Pairs of measurements for more than one category are shown in a scatterplot	0.007	0.007
A graph is not used to illustrate relative amounts	0.007	0.007
Animation is absent when it could be used to direct attention to a complex topic	0.000	0.000
A horizontal bar graph is not used when labels are too long to fit under a vertical display	0.000	0.000
All parts of static 3D diagrams are not shown from the same viewpoint	0.000	0.000

### Materials and methods

For all searching and coding, Mac OSX and Microsoft Excel were used. We used the Google search engine for the sampling procedure.

We first acquired a random, stratified sample of PowerPoint^®^ slideshows from the web. Following this, we asked two judges to score each PowerPoint^®^ slideshow independently, using a checklist (see Table 1) to search for violations of specific rules of effective PowerPoint^®^ communication; these rules were special cases of the eight general principles discussed in the Introduction. As noted below, two additional judges evaluated the slideshows using slightly different criteria.

#### Search procedure

Before we present the results of our sampling, we need to explain how we produced the sample. To ensure that there was no potential for bias (that is, sampling non-representative slideshows), we randomly selected slideshows within each category. To this end, we implemented a two-step procedure. We first constructed a list of keywords for each of four categories – Research (academic), Education, Government, and Business – by searching an electronic resource (e.g., EbscoHost) for the category name and then noting the keywords of the first few academic articles to appear in the search results. We then randomly paired each keyword with two numbers, which ranged from 1 to 10. The first number designated the page, the second the position of the item within a page. For example, a keyword, page, and position combination could have been “marketing, 6, 4.”

We then performed each search by entering the keyword followed by a space and then “.ppt” (the common abbreviation for PowerPoint^®^ slideshows). For example, if the keyword and page combination was “marketing, 6, 4,” the search term would have been “marketing.ppt,” which would yield a series of Google hits from which we would select the forth hit on the sixth page. In cases where the specified hit was not a slideshow, the next slideshow down would be selected, and if that entry was not a slideshow, we looked at the subsequent one, repeating this process until we found a slideshow. The minimum required length for slideshows was 15 slides, and the maximum allowed length was 100 slides; if slideshows contained fewer than 15 or more than 100 slides, we again selected the next slideshow in the list until we found a slideshow of suitable length.

In total, we selected 141 slideshows using this method, one of which was excluded because it clearly bridged categories, leaving us with a total of 140. We then classified each of these slideshows into one of five categories (in addition to Research, Education, Government, and Business, we defined a fifth category, Miscellaneous, for slideshows that did not fit clearly into the other four). Because we selected search terms that were targeted for a specific category, we were likely to find slideshows in that category – but our search method did not guarantee this result, and thus we double-checked the appropriate category after each slideshow was retrieved. Our search methods were designed to have a high probability of sampling from the different categories, and we had hoped to sample about the same number of slideshows from each category. However, our criteria were not perfect, and thus the numbers in each category are not precisely the same. Ultimately each category contained between 20 and 40 slideshows, specifically: Research: 27, Education: 38, Government: 20, Business: 32, and Miscellaneous: 23.

We note that we did not search for “.pptx” or “.key” or for other extensions. At the time the study was begun (2008), .pptx had not been available very long – and hence we worried about possible biases of sampling works from “early adopters.” In addition, the vast majority of slideshows are created with PowerPoint^®^, and we worried that Keynote^®^ users might also represent a biased sample. Thus, we can only be confident that our results generalize to PowerPoint^®^ slideshows *per se*. In addition, because we sampled from the web, our results can only generalize to other slideshows of the sort that are posted on the web (however, for our purposes, this is sufficient – if anything, these slideshows may be better than those not deemed worthy of being posted).

#### Scoring and coding

Four judges scored the 140 slideshows for violations of the 137 rules. Two of the judges were authors, and two were college-educated paid research assistants in the laboratory; at the time of coding, none of the judges knew whether or not they would subsequently do enough work on this project to be included as authors, and none were biased to find flaws (let alone flaws of specific types). Each rule was worded as a statement that, if it described a slide or slideshow as a whole, revealed a violation of that rule.

Two of the judges began by independently scoring 10 slideshows, and then comparing their scores. Any disparities were discussed, and in five cases the wording of rules was modified to be more specific. Following this initial training and calibration procedure, each judge independently scored all of the slideshows. The judges then discussed any disparities in classification or scoring, and reached a consensus.

The initial two judges interpreted each rule literally (“strictly”). For example, if the rule stated that no more than two lines should be included in a single bullet point, then a bullet point that had two lines and a single word in a third line would be classified as violating the rule. We adopted this procedure because we wanted to be sure that the rules could be easily interpreted. Nevertheless, we were concerned that we would inflate the number of violations by adhering rigidly to the rules; many of them were intended to be heuristics. For example, the prohibition against more than two lines per bullet point is based on the idea that no more than four concepts should be held in working memory at the same time, and in general two lines of text convey about four concepts.

Thus, following the initial scoring, two additional judges considered each of the violations identified by the first two judges. They decided, independently, whether each violation was important. In this case, “important” was defined as “likely to disrupt the comprehension or memory of the material.” If not, then they rejected that initial classification of a violation. By using both the strict and liberal scoring methods, we thereby defined a range in which we evaluated the slideshows. Table 1 shows the proportion of slideshows that violated each of the 137 rules, sorted by principle, scored separately for the strict and liberal criteria. Finally, two authors independently evaluated the rules and – using the summary provided in the introduction – indicated which principle applied to each rule. When they disagreed (for 15% of the rules), a discussion resolved the classification. In the process of this discussion, it became clear that 12% of the rules were “over-determined”: rather than following primarily from a single principle, they followed from two or more principles. Thus, we created a ninth level for the “principle” variable, which included all the rules for which more than one principle applied.

### Results

The dependent variable was whether or not a slideshow violated a principle. We took a “bad-apple-spoils-the-barrel” approach: if even a single slide contained material that violated a rule, the slideshow was scored as having violated the rule. And if a slideshow violated one or more rules within a specific principle, the score for that principle would be “1;” if none of the rules were violated for a principle, the score for it would be “0.” We present first the results from the strict, initial scoring, and after this the results from the more liberal scoring.

#### Strict scoring

We began by assessing inter-rater reliability. The overall inter-rater agreement was 0.88, which indicates the proportion of times that the raters either both coded “1,” indicating a violation, or both coded “0,” indicating no violation. We then sought to answer three questions about the data.

First, we asked whether the violations of the principles differed for the different categories (see Table 2). To answer this question, we conducted a repeated-measures ANOVA, with the nine levels of the Principles (the eight principles plus an “over-determined” class) as a within-participants variable and Category as a between-participants variable. The results showed that, in general, there were no overall differences in violations for the different categories, *F*(4, 135) = 2.24, *p* > 0.05, $\eta_{p}^{2}=0.06.$

**Table 2:** **Proportion of violations for each principle in each category for Study 1**.

Category	Principle	*N*	Strict scoring		Liberal scoring	
			Mean	SD	Mean	SD
Business	Appropriate knowledge	32	0.81	0.40	0.72	0.46
	Compatibility	32	0.53	0.51	0.41	0.50
	Discriminability	32	1.00	0.00	0.97	0.18
	Informative change	32	0.91	0.30	0.91	0.30
	Limited capacity	32	1.00	0.00	1.00	0.00
	Perceptual organization	32	0.50	0.51	0.50	0.51
	Relevance	32	0.88	0.34	0.81	0.40
	Salience	32	0.78	0.42	0.75	0.44
	“Over-determined”	32	0.56	0.50	0.53	0.51
Research	Appropriate knowledge	27	0.74	0.45	0.67	0.48
	Compatibility	27	0.15	0.36	0.11	0.32
	Discriminability	27	1.00	0.00	1.00	0.00
	Informative change	27	0.85	0.36	0.74	0.45
	Limited capacity	27	1.00	0.00	1.00	0.00
	Perceptual organization	27	0.26	0.45	0.22	0.42
	Relevance	27	0.74	0.45	0.74	0.45
	Salience	27	0.70	0.47	0.67	0.48
	“Over-determined”	27	0.41	0.50	0.37	0.49
Government	Appropriate knowledge	20	0.50	0.51	0.40	0.50
	Compatibility	20	0.40	0.50	0.35	0.49
	Discriminability	20	1.00	0.00	0.95	0.22
	Informative change	20	0.90	0.31	0.85	0.37
	Limited capacity	20	1.00	0.00	1.00	0.00
	Perceptual organization	20	0.40	0.50	0.40	0.50
	Relevance	20	0.75	0.44	0.70	0.47
	Salience	20	0.65	0.49	0.65	0.49
	“Over-determined”	20	0.35	0.49	0.35	0.49
Education	Appropriate knowledge	38	0.74	0.45	0.61	0.50
	Compatibility	38	0.26	0.45	0.26	0.45
	Discriminability	38	1.00	0.00	1.00	0.00
	Informative change	38	1.00	0.00	0.89	0.31
	Limited capacity	38	1.00	0.00	1.00	0.00
	Perceptual organization	38	0.34	0.48	0.34	0.48
	Relevance	38	0.76	0.43	0.76	0.43
	Salience	38	0.76	0.43	0.71	0.46
	“Over-determined”	38	0.32	0.47	0.29	0.46
Miscellaneous	Appropriate knowledge	23	0.61	0.50	0.43	0.51
	Compatibility	23	0.22	0.42	0.22	0.42
	Discriminability	23	1.00	0.00	0.96	0.21
	Informative change	23	0.96	0.21	0.87	0.34
	Limited capacity	23	1.00	0.00	1.00	0.00
	Perceptual organization	23	0.30	0.47	0.30	0.47
	Relevance	23	0.91	0.29	0.83	0.39
	Salience	23	0.87	0.34	0.83	0.39
	“Over-determined”	23	0.09	0.29	0.09	0.29

**Figure 1:**
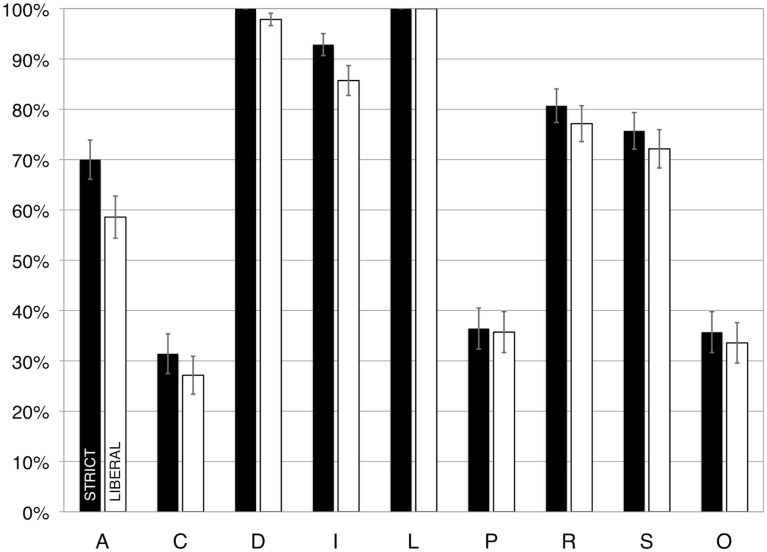
**The percentage of presentations that had violations, scored according to strict (dark bars) and liberal (light bars) criteria, in Study 1**. A, appropriate knowledge; C, compatibility; D, discriminability; I, informative change; L, limited capacity; P, perceptual organization; R, relevance; S, salience; O, over-determined. Error bars illustrate the standard error of the mean.

Second, we asked whether some principles were violated more frequently than others. As illustrated in Figure 1, in the same two-way ANOVA just noted, we found that some principles were violated more often than others, *F*(8, 1080) = 87.94, *p* < 0.01, $\eta_{p}^{2}=0.39.$ A Bonferroni-corrected *post hoc* test with an alpha of 0.05 revealed that out of the 36 possible comparisons, only the following eight were *not* significant: the difference between Appropriate Knowledge and Salience, Compatibility and Over-determined, Compatibility and Perceptual Organization, Discriminability and Limited Capacity, Informative Change and Relevance, Informative Change and Salience, Over-determined and Perceptual Organization, and Relevance and Salience. All other comparisons were significant.

In addition, the results of this analysis showed there was an interaction between Principles and Category, *F*(32, 1080) = 1.91, *p* < 0.05, $\eta_{p}^{2}=0.054.$ Specifically, a Bonferroni-corrected *post hoc* procedure documented that the difference in violations between the principle of Appropriate Knowledge and the principle of Compatibility was significantly different between the categories of Research and Government, *F*(1, 45) = 10.19, *p* < 0.01, $\eta_{p}^{2}=0.19.$

In general, the following aspects of these results are worth noting: the principles of Discriminability and Limited Capacity were violated in every single slideshow, and the principle of Informative Change was violated in 93% of the slideshows. In contrast, the principle of Compatibility was violated in only 31% of the slideshows.

Third, we examined which rules were violated most often. In particular, we found that the following five rules were violated most often: (1) Bulleted items are not presented individually, growing the list from the top to the bottom (96% of the slideshows); (2) More than two lines are used per bulleted sentence (91%); (3) More than four bulleted items appear in a single list (91%); (4) Hierarchical organization of lists is not used, with no more than four items at each level (86%); (5) All uppercase, all italics, or all bold typefaces are used (81%). To examine this result more carefully, we examined whether the five worst rules were violated equally often for the different categories of slideshows. The results showed no interaction between Category and the violation of the five worst rules, *F*(16, 540) = 1.51, *p* > 0.05, $\eta_{p}^{2}=0.04.$ See Table 3 for a more detailed breakdown of the worst rules per category.

**Table 3:** **Most frequently violated rules per category (strict scoring) for Study 1**.

Category	Rule	Proportion violated
Business	Bulleted items are not presented individually, growing the list from the top to the bottom	0.97
	More than four bulleted items appear in a single list	0.97
	Hierarchical organization of lists is not used, with no more than four items at each level	0.94
	More than two lines are used per bulleted sentence	0.81
	All uppercase, all italics, or all bold typefaces are used	0.78
Research	Bulleted items are not presented individually, growing the list from the top to the bottom	1.00
	More than two lines are used per bulleted sentence	1.00
	More than four bulleted items appear in a single list	0.85
	Hierarchical organization of lists is not used, with no more than four items at each level	0.81
	Words are not large enough (i.e., greater than 20 point) to be easily read	0.70
	Visual or auditory characteristics change even when they do not signal a change in information	0.70
Government	Bulleted items are not presented individually, growing the list from the top to the bottom	0.95
	All uppercase, all italics, or all bold typefaces are used	0.95
	More than two lines are used per bulleted sentence	0.95
	More than four bulleted items appear in a single list	0.95
	Hierarchical organization of lists is not used, with no more than four items at each level	0.90
Education	Bulleted items are not presented individually, growing the list from the top to the bottom	0.89
	More than two lines are used per bulleted sentence	0.89
	More than four bulleted items appear in a single list	0.89
	Visual or auditory characteristics change even when they do not signal a change in information	0.84
	All uppercase, all italics, or all bold typefaces are used	0.82
	Hierarchical organization of lists is not used, with no more than four items at each level	0.82
Miscellaneous	Bulleted items are not presented individually, growing the list from the top to the bottom	1.00
	More than two lines are used per bulleted sentence	0.96
	More than four bulleted items appear in a single list	0.91
	All uppercase, all italics, or all bold typefaces are used	0.87
	Hierarchical organization of lists is not used, with no more than four items at each level	0.83

#### Liberal scoring

We again assessed inter-rater reliability, now based on the judgments with the more liberal criteria. The proportion agreement between the two raters for all principles and scales combined was 0.98. The shift from strict to liberal criteria did not change the pattern of the results. Using the new criteria resulted in our eliminating only 4% of the violations that were identified using the strict scoring procedure.

Considering again our three questions about the data: first, as with the strict coding, we conducted a repeated-measures ANOVA, with the nine levels of the Principles, as a within-participant variable, and Category as a between-participant variable. The results showed that, in general, there were no differences in violations between the categories, *F*(4, 135) = 1.93, *p* > 0.1, $\eta_{p}^{2}=0.05.$

Second, we again asked whether some principles were violated more often than others. As illustrated in Figure 1, we found that some principles were violated more often than others, *F*(8, 1080) = 79.52, *p* < 0.05, $\eta_{p}^{2}=0.37.$ A Bonferroni-corrected *post hoc* test with an alpha of 0.05 showed that out of the 36 possible comparisons, only the following eight were *not* significant: the difference between Appropriate Knowledge and Salience, Compatibility and Over-determined, Compatibility and Perceptual Organization, Discriminability and Limited Capacity, Informative Change and Relevance, Informative Change and Salience, Over-determined and Perceptual Organization, and between Relevance and Salience. All other comparisons were significant.

As before, the principles of Discriminability (98%), Limited Capacity (100%), and Informative Change (86%) each were violated in the vast majority of slideshows. Also as before, the principle of Compatibility was violated least-frequently, in “only” about a quarter of the slideshows (27%) in this case. The results of the analysis also showed that there was no interaction between Category and Principle, *F*(32, 1080) = 1.44, *p* > 0.05, $\eta_{p}^{2}=0.04.$

Third, we again examined which rules were violated most often. In this analysis, we found that the following five rules were violated most often: (1) Bulleted items are not presented individually, growing the list from the top to the bottom (94% of the slideshows); (2) More than four bulleted items appear in a single list (88%); (3) More than two lines are used per bulleted sentence (87%); (4) Hierarchical organization of lists is not used, with no more than four items at each level (84%); (5) Words are not large enough (i.e., greater than 20 point) to be easily seen (65%). The first four rules are the same as the first four identified in the previous analysis, but now the rule “All uppercase, all italics, or all bold typefaces are used” was replaced by another rule that also stemmed from the principle of Discriminability, namely the rule regarding size. To examine this result more carefully, we examined whether there was an interaction between the five worst rules and categories, and found such an interaction, *F*(16, 540) = 2.35, *p* < 0.05, $\eta_{p}^{2}=0.07.$ Specifically, a Bonferroni-corrected *post hoc* procedure showed that the difference in violations between the rule “Words are not large enough (i.e., greater than 20 point) to be easily seen” and the rule “More than two lines are used per bulleted sentence” was significantly different between the categories of Business and Government, *F*(1, 50) = 10.99, *p* < 0.01, $\eta_{p}^{2}=0.18.$

Table 2 provides a summary of the violations per category per principle, using both the liberal and the strict scoring method.

### Conclusion

As is clear, by either the strict or liberal scoring criteria, many slideshows are flawed. Indeed, not a single slideshow was scored – according to either set of criteria – as having no flaws. Using the strict criteria, each slideshow violated on average 6.23 of the nine classes of principles, which shrank slightly to 5.88 using the liberal criteria. Considering just the eight individual principles, excluding the “over-determined” group of rules, the overall violation was 6.17 for the strict coding and 5.86 for the liberal coding. The three most-violated principles were Discriminability (because material was too similar to be easily distinguished), Limited Capacity (because too much information was presented), and Informative Change (because changes in how information was presented did not actually reflect changes in the information being conveyed). Even the least-frequently violated principle, Compatibility, was still violated in at least a quarter of the slideshows.

In addition, the fact that violations were comparable across the different categories is of interest. We examined the different categories partly in an effort to consider the possible influence of different topics. Although slideshows of different topics (or areas) may differ in their average complexity, the results do not suggest that violations of specific principles generally vary for different topics. That said, we did find that some specific rules were violated more frequently in some categories than others, which could reflect differences in the complexities of the topics.

In sum, the present results suggest that the psychological principles either are not obvious or are obvious but often ignored.

## Study 2

The analysis of PowerPoint^®^ slideshows in Study 1 revealed that slideshows themselves typically are flawed in multiple ways. We wanted to know whether viewers are sensitive to presentation flaws more generally, and thus we conducted a survey asking participants to report approximately how many electronic slideshow presentations they had seen in the past year, and then to rate various aspects of those presentations in terms of their quality.

### Materials and methods

#### Participants

We recruited 265 participants via ads on Craigslist and by word-of-mouth. In order to participate in the study, participants had to be at least 18 years old, speak fluent English, and “regularly” attend presentations that involved PowerPoint^®^, Keynote^®^, or other electronic slideshows. (In order to receive compensation – either a $5 gift certificate to Amazon.com or a free book – they had to be US citizens or permanent residents and not employed by Harvard University.) All participants provided their informed consent and were tested in accordance with the ethical guidelines of the American Psychological Association; this research was approved by the Harvard University Committee on the Use of Human Subjects.

Forty-six of the participants did not meet the eligibility criteria of viewing at least one presentation per month during the previous year, and 14 skipped more than 20% of the questions. Therefore, analyses were conducted on data from the 205 eligible participants who completed at least 80% of the survey, including 112 females (mean age 32.5 ± 8.9) and 83 males (mean age 30.9 ± 8.9) and 10 who did not report their sex or age. According to self-report, about 7% of the participants were engineers, 10% were in finance, 13% were business managers or executives, and 14% were students; the remaining participants were distributed across a variety of different occupations.

#### Materials

The study was administered over the internet via a secured link (SSL) on Surveymonkey.com. The opening page of the survey consisted of a statement about the eligibility requirements and a description of the study content. Participants were assured that their data would be collected anonymously, and that they would be sent to another unrelated survey in order to provide the personal information (email address and physical address) we needed in order to send the gift certificate.

The survey began by asking approximately how many electronic slideshow presentations the participant viewed each year (up to “5 or more per week”). The main portion of the survey consisted of a series of questions about electronic slideshow presentations viewed in the last year. We asked two sorts of questions. First, we asked about the presence of specific problems; for example, we asked “Did any of the presentations fail to convey a meaningful message because there was no main point?” and “Were any of the presentations hard to follow because the slides contained too much material to absorb before the next slide was presented?” Participants were asked to indicate the proportion of presentations – if any – that had such problems, using the following scale: “None (of the presentations); Some; Half; Many; Virtually all.” Second, we asked questions about annoyance, querying the degree to which the problem, if present, bothered them; these responses used a four-point scale, labeled as follows: “Not at all; Somewhat; A fair amount; It was extremely annoying to have my time wasted in this fashion.” It could be argued that by focusing on problems, rather than framing the questions in a neutral fashion, we were biasing the participants to make negative responses – and this is a possible limitation of the survey. However, it is easier, grammatically and logistically, to ask people to notice problems than to notice things that were flawless. If a presentation is excellent the audience will be focused on the presenter’s message, not on the technicalities of the presentation.

The questions covered typical violations of seven of the eight principles. Because we were asking for overall impressions across all presentations recalled from the past year, rather than analyses of specific slides, we did not ask any questions about Perceptual Organization, fearing that participants would not be able to recall these types of details accurately. We included 12 questions about Relevance; 8 about Appropriate Knowledge; 4 about Salience; 5 about Discriminability; 3 about Compatibility; 2 about Informative Change; 7 about Limited Capacity; and one that did not clearly fit into any of these categories, “Were any of the speakers unprepared in that they didn’t know how to use their technology (software, computer, projector, etc.)?” We also included “free response” questions, asking participants to report any other common problems *and* any techniques that they thought contributed to especially effective presentations. The final portion of the survey asked for basic demographic information, including age, sex, and occupation (which they could provide explicitly or select from a checklist, or both).

### Results

The Appendix presents a table with all of the questions, the average ratings, and the percent responses in each category of the rating scale, for both prevalence and annoyance. The five items with the highest ratings (bold and underlined) and the five with the lowest (italics) for Prevalence and Annoyance are highlighted in gray. Note that one of the most prevalent violations (lack of a pointer) was one of the least annoying.

We coded the data in two ways. First, we converted the five “prevalence of violation” and four “annoyance” ratings into numerical values (0–4, and 0–3, respectively), and then for each participant we calculated the average rating for prevalence of violations and for associated annoyance level for each principle. Because the most common answers were “none” and “some,” we also coded the data using a binary coding system, coding any degree of violation [“some (presentations),” “half,” “many,” “virtually all”] as “1,” and “none” as “0,” and any degree of annoyance (“somewhat,” “a fair amount,” “it was extremely annoying to have my time wasted in this fashion”) as “1,” and “not at all” as “0,” and then again for each participant calculated an average score for both prevalence and annoyance for each of the principles. (The fact that these were the most common answers suggests that participants were not biased by our questions.)

As in Study 1, we asked whether some principles were violated more often than others, calculating one-way repeated-measures ANOVAs. For all ANOVAs Mauchly’s test indicated that the assumption of sphericity had been violated (chi-square > 163, *p* < 0.0005, epsilon > 0.75), therefore degrees of freedom were corrected using Huynh–Feldt estimates of sphericity. The results were similar regardless of the coding used (full scale or binary), revealing that some of the principles were violated more often than others, *F*(4.5, 927) = 10.80, *p* < 0.0005, $\eta_{p}^{2}=0.05$ for the full scale, and *F*(4.9, 999) = 8.00, *p* < 0.0005, $\eta_{p}^{2}=0.04$ for the binary scale. Overall, respondents reported that all seven principles were violated at least some of the time (see Table 4). These reports ranged from a low of 63% of respondents reporting at least some presentations with violations of Compatibility to a high of 74% reporting at least some with violations of Salience.

**Table 4:** **Percentage of respondents reporting “at least some” prevalence of violations and associated annoyance, by principle, in Study 2**.

Principle	Prevalence	Annoyance
Appropriate knowledge	72.5	65.2
Compatibility	62.5	60.5
Discriminability	65.4	65.0
Informative changes	67.3	58.6
Limited capacity	70.1	65.0
Relevance	68.4	64.6
Salience	73.5	62.2

We also asked whether violations of some principles were more annoying than violations of other principles. (Data from 197 participants, rather than 205, were used in these analyses, because some of those who reported no violations skipped the “annoyance” questions.) One-way repeated-measures ANOVAs revealed that violations of some principles were more annoying than others *F*(4.9, 968) = 5.15, *p* < 0.0005, $\eta_{p}^{2}=0.03$ (full scale), and *F*(4.7, 928) = 3.05, *p* < 0.05, $\eta_{p}^{2}=0.02$ (binary); these results are also presented in Table 4. The range was similar to the previous measure, but different principles anchored it: nearly 59% of respondents reported at least some annoyance with violations of Informative Change, whereas over 65% reported at least some annoyance with violations of Appropriate Knowledge.

Although we did document a significant difference in reported prevalence and annoyance for the violations of different principles, it was relatively small and the most interesting results may be in the responses to individual questions. The three most prevalent problems were also among the five most annoying: “Speakers read word-for-word from notes or from the slides themselves” (Salience), “The slides contained too much material to absorb before the next slide was presented” (Limited Capacity), and (most prevalent and most annoying), “The main point was obscured by lots of irrelevant detail” (Relevance).

Because different numbers of questions were asked for different principles, we conducted correlation analyses to discover whether the mean ratings were related to the number of questions per principle. No significant correlations were found between prevalence or annoyance ratings and the number of questions per principle, nor were prevalence and annoyance significantly correlated with each other.

We also invited the participants to make free responses about presentation problems that were not covered in the survey, and to indicate key aspects of especially good presentations. These free responses, in general, tended to reiterate topics covered in the survey, for instance, “When explaining complicated information, it’s important to use as many slides as necessary. Do not try and cram it all together.” Or, “a little humor goes a long way.” However, many respondents complained that speakers should be better prepared, by “rehearsing their lectures so they don’t look like it’s the first time they’re seeing these slides too,” and by “hav(ing) everything ready and working properly before (the audience) show(s) up” and coordinating carefully with any assistants who operate the equipment so that technical problems do not derail the entire talk. (About 61% of the respondents reported that at least some of the presentations were flawed because the presenter did not know how to use his/her equipment, and more than 50% of the respondents indicated that this was at least somewhat annoying.) In short, “Don’t waste my time! If you aren’t going to properly prepare just send me a memo with information instead!”

### Conclusion

These results converge with those from the Study 1, namely that all seven principles we asked about (out of eight considered in the first study) are violated on a regular basis. Such violations bother audience members to some extent. And as with Study 1, violations of the Principle of Compatibility were least common, whereas one of the most annoying and prevalent violations fell under the principle of Limited Capacity (slides contained too much material to absorb before the next slide was presented). These results show that presentations not only have much room for improvement, as shown in Study 1, but also that audience members are sensitive to the general sorts of problems that plague presentations.

The findings from Study 1 and Study 2 thus raise an interesting question: why do at least some people feel that the psychological principles are so obvious that nobody would violate them? Is it possible that although people recognize overall problems – such as that a presentation is boring or unclear – they fail to recognize the precise problems with specific slides that produce such easily noticed flaws? We investigate this possibility in the following study.

## Study 3

The present study was designed to answer two questions: first, we wanted to know whether people can identify graphical displays in individual slides in which the different principles have been violated. Second, if they can identify such defective displays, we wanted to know whether participants are aware of *why* a display is defective.

The previous two studies were surveys. Such studies have the advantage of tapping into phenomena that occur in the “real world,” but the disadvantage of not having rigorous control over the relevant variables. Study 3 relies on an experiment. We selected a subset of the visual display design rules from Study 1, and prepared two illustrations for each one: one illustration included a violation of the rule and one did not (many of these pairs were taken directly from the “Do/Don’t” illustrations shown in Kosslyn, 2007). Prior to presenting each pair of slides, we presented a question that guided participants to focus on a specific aspect of the slide. For example, for one slide pair we asked, “Which slide does a better job showing how closely two variables are related?” Participants were asked to indicate which slide was better, according to that specific criterion, and then to explain why they made their choice.

### Materials and methods

#### Participants

A total of 53 participants took part in Study 3; however, five of these participants did not follow the instructions, so their data were not included in the analyses. The analyzed data came from 21 males with a mean age of 29.3 (SD = 11.1) and 27 females with a mean age of 23.6 (SD = 5.3). Participants were recruited through a Harvard University study pool, which was open to Harvard affiliates and members of the Cambridge/Boston community. None of the participants had prior knowledge of the presented material. All participants provided their informed consent and were tested in accordance with the ethical guidelines of the American Psychological Association; this research was approved by the Harvard University Committee on the Use of Human Subjects.

#### Materials

Forty-one “Do/Don’t” pairs of illustrations from Kosslyn (2007) formed the core of our stimulus pairs; we removed the “Do/Don’t” labels from these illustrations. We created 23 additional pairs of illustrations (some from Kosslyn, 2006), to ensure that we had adequate examples for each of the eight principles. Four pairs were eventually eliminated because of problems with the quality of the illustrations, leaving us with six pairs of illustrations for Appropriate Knowledge, seven each for Perceptual Grouping and Salience, and eight for each of the other principles. We randomized the presentation order across principles, with the constraint that two stimuli in a row could not probe the same principle. We then randomly put the version with the violation on the left for half of the slides, and on the right for the other half. No more than five slides in a row had the violation on the same side of the pair. We next created another set of stimuli by reversing the left and right panels for each pair (and thus, for each pair the violation appeared on the left in one version, and on the right in the other). Finally, we created two orders, forward and reverse, for each of the two stimulus sets, which resulted in a set of four stimulus sequences.

In addition, we prepared a question for each pair of illustrations. Each question asked participants to choose a panel (left or right) of the pair according to a particular criterion, such as: “Which slide does a better job of using textures?”

We tested groups of 3–10 participants together. Participants met in a computer lab, and each was seated at his or her own computer (Apple iMacs, with 20″ screens). We administered each of the four sets of stimuli equally often, and thus counterbalanced for presentation order (left/right and forward/reverse).

**Figure 2:**
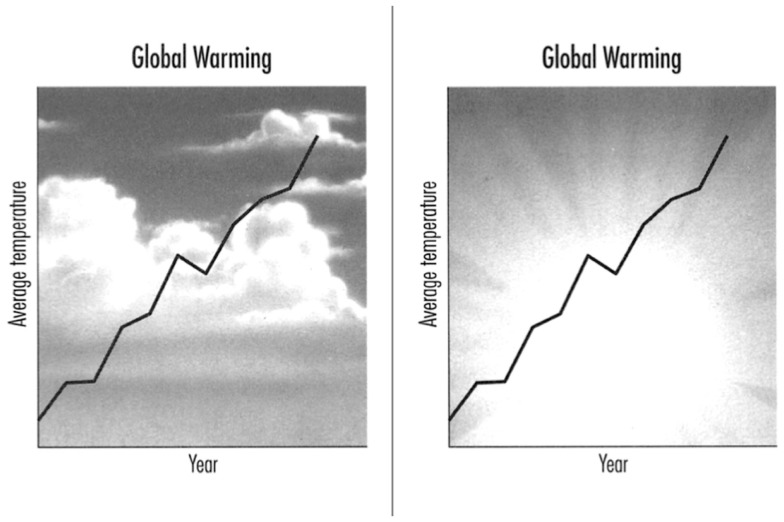
**Example of a slide pair viewed by participants in Study 3 (illustrating the principle of Compatibility)**. Prior to seeing this pair, they received the question, “Which slide does a better job of using a background pattern?” The correct answer for slide choice is the panel on the right. An example of a correct explanation is, “the topic of the chart is global warming, which is better represented with a sun than with clouds;” an example of an incorrect explanation is, “the right has more of an apocalyptic feel.”

On each trial, participants first viewed the question and then the accompanying pair of illustrations. Participants indicated each choice by typing their answer (“l” or “r”) in a column in a spreadsheet. After choosing, the participants typed a brief explanation of their choice. The participants viewed the questions and pairs of stimuli at their own pace. We include examples of participant responses below, in addition to Figure 2, which illustrates a stimulus pair.

For instance, we asked the question, “which slide does a better job using the key (the labeled squares at the top)?” The same bar graph was used in both slides. The better slide ordered the patches (squares) in the key to correspond with the order of the bars on the graph (which had the darkest bar in the middle); the flawed slide ordered the patches from lightest to darkest. A correct explanation for selecting the correct slide (matching order) was, “the key is laid out in the same sequence as the bars on the graph.” An incorrect explanation for selecting the correct slide (matching order) was, “the patterns in the key are arranged more dynamically with better contrast.” A completely incorrect response was to choose the non-matching order “because the key feels more orderly, the lightest shade on top and the darkest shade on the bottom.”

On another trial we asked, “Which slide uses the background pattern more effectively?” The two slides contained identical captioned and labeled line graphs with home prices over time; both had a photo of a house in the background. In the flawed slide, the background photo was inappropriately pronounced, making the graph difficult to read. The better slide used the same photo, but as a de-saturated watermark; it was visually interesting without in any way obscuring the graph. A correct explanation for selecting the correct slide (graph more salient than background) was, “[the background] is subtle but nice and doesn’t overshadow the graph.” An incorrect explanation for the correct slide (graph more salient) was, “it is more explanatory.” A completely incorrect response was to choose the slide with the too-salient background because, “you can see all of the beautiful scenery behind the home as well as the attractiveness of its surroundings.”

### Results

**Figure 3:**
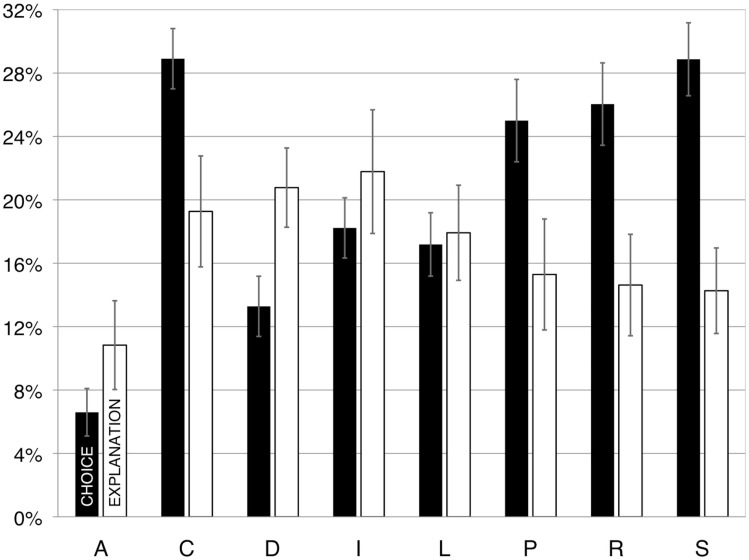
**The percentage of trials on which participants made errors in choosing the correct slide (dark bars), and explaining their choice (light bars) given that they chose the correct slide, in Study 3**. A, appropriate knowledge; C, compatibility; D, discriminability; I, informative change; L, limited capacity; P, perceptual organization; R, relevance; S, salience. Error bars illustrate the standard error of the mean.

Because we asked the participants not only to choose one member from each pair, but also to explain their choice, we computed two types of scores: the error rate for *slide choice* and for *explanation*. Two judges (one of whom is an author of this article, and the other of whom was a paid research assistant in the lab) independently scored the explanations participants provided for each of their choices. These judges determined whether the participant had in fact identified the key feature that distinguished the versions of the illustration. We used “liberal” criteria, giving the participants the benefit of the doubt if there were any questions about their accuracy. Inter-rater agreement for the two judges was substantial, at 0.90 (ranging from a low of 0.87 for the principle of Perceptual Grouping to a high of 0.93 for the principle of Relevance). When the judges disagreed in their initial scoring, they discussed the item in question and came to a consensus agreement. Table 5 and Figure 3 present the results for slide choice and explanation organized according to principle.

**Table 5:** **Percentage of incorrect slide choices and incorrect explanations of correct choices, by principle, in Study 3**.

Principle	Incorrect choices	Incorrect explanations following correct choice
Appropriate knowledge	6.6	10.8
Compatibility	28.9	19.3
Discriminability	13.3	20.8
Informative changes	18.2	21.8
Limited capacity	17.2	17.9
Perceptual organization	25.0	15.3
Relevance	26.0	14.6
Salience	28.9	14.3

The mean error rate for slide choice was 20.5% (with chance performance at 50% errors). A repeated-measures ANOVA revealed that the error rates for slide choice varied for the different principles, *F*(7, 329) = 22.60, *p* < 0.0005, $\eta_{p}^{2}=0.325.$ As is evident in Table 5 and Figure 3, the participants were most accurate for the principle of Appropriate Knowledge and least accurate for the principles of Compatibility and Salience. Why did these participants have such a problem with Compatibility, when this principle is the least violated in the other studies? A close look at the results indicated that the present finding is driven by rules for very specific types of displays that may not be used frequently (and thus may not have been encountered in the presentations sampled in Study 1 or queried in Study 2).

The five stimulus pairs that induced the most errors in slide choice illustrated the following rules: explode a max of 25% of wedges in a pie (Salience); use a line graph to display interactions over two entities on the *X* axis (Compatibility); use different colors only for emphasis or to specify different classes of information (Informative Change); use a scatterplot to convey an overall impression of the relationship between two variables (Compatibility); use one color for titles and another, less salient one for text (Salience).

Although the participants chose the correct alternative on about 80% of the trials, on fully one sixth of these trials, they could not articulate why they made these choices; that is, 16.8% of the explanations for correct choices were incorrect. Thus, for only about two thirds of the total trials did they choose correctly on the basis of knowledge. A repeated-measures ANOVA showed that the participants made more errors for some principles than others when explaining their choices, *F*(7, 279) = 3.47, *p* = 0.003, $\eta_{p}^{2}=0.069.$ (Mauchly’s test indicated that the assumption of sphericity had been violated: chi-square = 59, *p* < 0.0005, epsilon = 0.847, therefore degrees of freedom were corrected using Huynh–Feldt estimates of sphericity.) The participants made relatively more errors when explaining violations of the principles of Discriminability and Informative Change, and were relatively accurate when explaining their choices for the principle of Appropriate Knowledge. Participants had the most difficulty explaining their (correct) choices for displays that illustrated the following rules: avoid using red and blue in adjacent regions (Discriminability); use hierarchical labeling (Limited Capacity); ensure that shapes of meaningful regions are easily identifiable (Discriminability); use one color for titles and another, less salient one for text (Salience); graph different types of data in a single display only if they are highly related and must be compared (Relevance).

Finally, Table 6 presents the percentage of participants who made *at least one error* in slide choice per principle, and (given they made the *correct* slide choice) *at least one error* in explanation per principle; note that there are no principles for which there are no errors.

**Table 6:** **Percentage of participants who made *at least one error* in slide choice per principle, and percentage of participants who (given they made the correct slide choice) made *at least one error* in explanation per principle, in Study 3**.

Principle	Slide choice	Explanation
Appropriate knowledge	33.3	39.6
Compatibility	97.9	54.2
Discriminability	62.5	85.4
Informative changes	85.4	60.4
Limited capacity	75.0	62.5
Perceptual organization	81.3	45.8
Relevance	87.5	45.8
Salience	89.6	47.9

### Conclusion

In short, even when guided to pay attention to specific features of slides, observers do not always understand when the psychological principles have been violated, and even when they do realize that there is a problem, they often are not aware of the nature of this problem. Given the way information processing works in the brain, however, one could argue that even if viewers do not notice a violation, they could still be affected by it. Indeed, we would predict this to be the case, and it is well worth investigating this hypothesis in future research.

## Discussion

In the three studies reported here, we analyzed how well PowerPoint^®^ slideshows, slides, and presentations respect principles of human perception, memory, and comprehension. Specifically, we hypothesized and found that the psychological principles are often violated in PowerPoint^®^ slideshows across different fields (Study 1), that some types of presentation flaws are noticeable and annoying to audience members (Study 2), and that observers have difficulty identifying many violations in graphical displays in individual slides (Study 3). These studies converge in painting the following picture: PowerPoint^®^ presentations are commonly flawed; some types of flaws are more common than others; flaws are not isolated to one domain or context; and, although some types of flaws annoy the audience, flaws at the level of slide design are not always obvious to an untrained observer (but, we would argue, such flaws may negatively impact information processing). Specifically, in Study 1 we found that PowerPoint^®^ slideshows on average violate approximately six of the eight principles we considered. Moreover, this is true for slideshows produced in different fields. The principles of Discriminability, Limited Capacity, and Informative Change were most frequently violated. In Study 2 we found that respondents commonly reported having noticed and been annoyed by problems in presentations in general, and these problems arose from violations of most of the principles we considered. Finally, in Study 3 we showed that observers often did not recognize when a specific rule was violated in a given slide, and that even when they did select the slide without the violation, they often could not explain their choice.

But can we be confident that violations of our principles really constitute “flaws” in a presentation? As noted in the Introduction, our principles grow out of a straightforward task analysis, which in turn led us to consider basic findings in the scientific literature on human perception, memory, and comprehension. Because of this connection, the principles have face validity: if a presentation violates a principle, it taxes human information processing. And because the presentation taxes information processing, the audience members will have difficulty perceiving, remembering, or comprehending it – and hence, it can reasonably be said to be flawed.

Nevertheless, future studies should explicitly examine the effects of violating specific principles on comprehension and subsequent memory. Such studies should also assess the degree to which audience members enjoy presentations with specific flaws, and their ability to think critically about such presentations (both while they are underway and subsequently). In addition, future studies should address whether specific types of violations have the same effects for slideshows that are presented versus for slideshows that are posted or distributed (and hence meant to be understood independently of a presenter).

By the same token, one could ask whether the rules “measure what they are supposed to measure.” To be clear: the rules do not “measure” anything; we are not presenting a new psychometric instrument. The rules are guidelines that should be obeyed to avoid including characteristics that will impair information processing. In the current research, we devised rules to capture key characteristics that would make presentations difficult to perceive, remember, or comprehend. As such, the “psychometric properties” associated with such a checklist are inherently different from a formal psychological test. For example, the fact that a specific rule is violated by every single presentation is, in the current study, very informative – whereas this is not true for an item in a conventional psychological test.

It is worth noting that respecting the principles and rules we have described will not produce “optimal” presentations. Instead, respecting the principles and rules will ensure that presentations are not flawed in these specific ways. Simply avoiding the glitches that we document will not ensure that the presentation is good, but including such glitches will make a presentation bad (or at least not as good as it could be). Given that the principles and rules are rooted in the empirical literature, avoiding violating them will improve a presentation. (However, we also must note that this claim is based on face validity, and must be explicitly tested in subsequent work).

We also must point out that in two of the three studies reported here, we treated the slides independently of the presenter. One could argue that at least some of the potential “flaws” may not be as severe when the slides are discussed by a skilled presenter. For instance, a talented presenter may be able to compensate for a particular flaw (e.g., by reading aloud text that is so small as to be all-but-invisible for the audience members). However, all else being equal, there is no advantage – and considerable disadvantage – to showing flawed slides. Although some flaws may be compensated for by talented presenters, it would be better if they did not have to compensate in the first place.

In short, we have documented that there is plenty of room for improvement in the design of electronic slideshow presentations. The psychological principles apparently are not entirely obvious, nor do people always respect them instinctively. Perhaps the best way to avoid such flaws is to prepare a slideshow draft, review each slide according to the psychological principles and rules we describe here, and then correct flaws as they are detected. Presenters may also want to review the Appendix in order to avoid common presentation mistakes, particularly those that the audience reports as highly annoying. It is worth noting that presentation techniques designed to compensate for poorly designed slides (such as reading aloud slides with miniscule text), may sometimes backfire – causing the audience to lose interest and tune out. Making your audience do unnecessary work is not a recipe for a successful presentation.

## Conflict of Interest Statement

The authors declare that the research was conducted in the absence of any commercial or financial relationships that could be construed as a potential conflict of interest.

## Appendix

Study 2 survey questions, average ratings, and percent responses in each ratings category for *Prevalence* (“What proportion of presentations?” Ratings categories: 0 = “none”; 1 = “some”; 2 = “half”; 3 = “many”; 4 = “virtually all”) and *Annoyance* (“Did this bother you?” Ratings categories: 0 = “not at all”; 1 = “somewhat”; 2 = “a fair amount”; 3 = “it was extremely annoying…”).

The five items with the highest ratings (bold and underlined) and the five with the lowest (italics) for Prevalence and Annoyance are highlighted in gray.

**Table:**

		Average rating	Prevalence					Average rating	Annoyance			
			Percent responses in each rating category (0–4 scale)						Percent responses in each rating category (0–3)			

Principle	Item		0	1	2	3	4		0	1	2	3
**DID ANY OF THE PRESENTATIONS FAIL TO CONVEY A MEANINGFUL MESSAGE BECAUSE**												
R	There was no main point	1.039	28.8	48.3	14.6	5.4	2.4	1.170	32.7	28.8	17.6	15.6
R	The main point was obscured by lots of irrelevant detail	**1.363**	14.6	52.2	19.0	9.3	4.4	**1.340**	20.0	37.1	25.4	13.7
R	Not enough information was provided to support the main point	1.201	21.5	50.2	15.6	10.7	1.5	**1.274**	22.9	36.1	24.9	12.2
**DID THE SPEAKER EVER FAIL TO CONNECT WITH THE AUDIENCE BECAUSE HE OR SHE**												
A	Was talking about a topic that the audience did not care about	1.146	23.4	52.7	12.2	9.3	2.4	1.162	29.8	36.6	15.1	15.1
R	Did not explain the importance of the topic	1.063	29.8	45.4	14.6	9.3	1.0	1.046	27.3	42.4	20.0	5.9
A	Assumed the audience knew more than it did	1.205	26.3	43.9	15.6	11.2	2.9	1.015	34.6	34.1	20.5	7.8
A	Assumed the audience knew less than it did	1.166	25.9	48.3	12.7	9.8	3.4	1.096	32.2	31.7	23.9	8.8
A	Used too much jargon	1.249	22.9	48.3	12.7	13.2	2.9	1.060	28.3	40.0	24.4	4.9
A	Went through the presentation too fast	0.902	38.0	44.9	7.8	7.3	2.0	0.864	42.9	29.3	20.0	4.9
A	Went through the presentation too slowly	1.265	22.4	46.3	16.1	11.2	3.4	**1.262**	24.9	31.7	27.3	11.2
**WERE ANY OF THE PRESENTATIONS HARD TO FOLLOW BECAUSE THE SPEAKER**												
L	Did not follow a clear narrative structure (with a beginning, middle, and end)	0.985	29.8	52.7	6.8	7.8	2.0	0.865	32.2	45.4	12.7	3.4
A	Did not provide an overview of the talk in the introduction	0.995	33.2	46.8	8.8	8.3	2.4	*0.788*	41.0	35.1	15.1	2.9
S	Did not use his/her slides to provide direction to the talk	1.064	28.8	48.8	12.2	6.3	3.4	0.883	35.6	40.0	15.6	4.4
L	Did not use his/her slides to illustrate complex or critical information	1.044	28.8	50.2	10.7	6.8	2.9	0.953	32.2	39.5	17.1	5.4
S	Did not use a pointer or otherwise direct audience attention to important details on the slides	**1.279**	28.3	43.9	8.3	9.3	9.8	*0.680*	48.3	32.7	9.3	4.4
L	Did not provide a summary of his/her key points at the end	1.143	30.2	41.5	14.6	8.3	4.4	0.969	36.1	30.7	20.5	6.3
R	Did not have a clear conclusion	1.114	27.8	46.3	12.7	7.3	3.9	1.089	27.3	38.5	19.0	8.3
**WERE ANY OF THE PRESENTATIONS HARD TO FOLLOW BECAUSE THE SLIDES**												
L	Contained too much material to absorb before the next slide was presented	**1.356**	18.0	45.4	21.5	13.2	2.0	**1.225**	22.9	38.5	27.3	8.8
L	Had too many bullets	1.039	33.2	44.4	9.8	10.7	2.0	0.908	35.1	40.0	14.6	5.9
D	Had text or graphics that were too small to see clearly	1.171	24.4	50.7	10.7	11.7	2.4	1.195	25.9	34.6	29.3	7.8
D	Used colors or fonts that were hard to distinguish	0.932	34.1	49.3	6.8	8.8	1.0	1.031	35.1	30.7	21.5	8.3
R	Did not have enough graphs, pictures, or diagrams	0.932	33.2	50.7	8.3	5.4	2.4	0.854	37.6	38.5	17.6	2.9
C	Contained irrelevant graphics, backgrounds, or animations	0.980	36.6	42.4	11.2	5.9	3.9	1.005	36.6	29.3	22.4	7.3
I	Varied fonts or colors for no reason	1.029	33.2	46.8	7.8	8.3	3.9	0.842	40.0	35.6	15.1	4.9
L	Contained graphics that were too complex	*0.761*	43.9	41.0	11.2	2.9	1.0	*0.758*	43.4	34.1	13.7	3.4
D	Contained poor quality images or video	0.877	38.0	46.3	6.8	5.9	2.4	0.891	37.6	35.1	15.6	5.9
**WERE ANY OF THE PRESENTATIONS HARD TO UNDERSTAND BECAUSE THE SPEAKERS**												
D	Mumbled	0.824	38.5	47.3	7.3	5.4	1.0	1.063	34.6	28.3	21.0	9.8
D	Spoke too quietly/did not use the microphone properly	0.882	37.1	45.9	8.8	6.8	1.0	1.016	33.7	33.7	17.6	8.8
R	Did not answer questions	*0.719*	48.8	36.6	8.3	3.4	2.0	0.791	44.4	27.3	18.0	3.4
R	Allowed too many questions to interrupt the talk	0.931	39.5	40.0	11.2	4.9	3.9	0.932	39.0	27.3	20.0	6.3
L	Did not give the audience time to digest important points	1.119	23.4	53.2	12.2	6.3	3.4	1.045	30.2	38.0	22.0	6.3
C	Presented slides with a lot of text and then proceeded to talk while the audience was trying to read	1.255	23.9	45.9	13.7	12.7	3.4	1.116	28.8	37.1	21.5	9.3
**DID ANY OF THE SPEAKERS**												
S	Read word-for-word from notes or from the slides themselves	**1.348**	19.5	48.3	12.7	15.6	3.4	**1.315**	27.3	27.8	26.8	15.6
S	Talk to the screen rather than to the audience	1.059	28.8	47.3	15.1	5.4	2.9	0.974	33.2	36.1	19.0	5.9
A	Fail to pay attention to audience “feedback” (raised hands, puzzled looks, fidgeting, and signs of boredom)	1.142	27.8	43.4	18.0	6.8	3.4	1.081	31.7	31.7	25.9	6.8
R	Not inject any humor or illustrations to lighten complex material	**1.299**	23.9	43.4	15.1	12.7	4.4	0.899	35.6	38.5	19.0	3.4
C	Use inappropriate or irrelevant jokes or illustrations	*0.631*	50.7	39.5	5.4	1.5	2.0	*0.691*	47.8	28.8	14.1	2.4
**WERE ANY OF THE SPEAKERS UNPREPARED, IN THAT THEY**												
R	Stumbled over their presentation, repeated themselves, seemed puzzled by their own slides	*0.751*	42.4	43.9	10.2	2.9	0.5	0.867	41.0	34.1	12.7	7.8
I	Had mistakes in their slides	0.907	32.2	52.7	8.3	5.9	1.0	0.862	39.0	33.7	19.0	3.4
R	Went over the allotted time (or rushed to finish in time)	1.186	25.9	44.9	15.1	11.7	2.0	1.117	27.3	39.5	20.0	9.3
R	Did not have enough material to fill the time	*0.815*	41.0	45.4	6.8	4.9	2.0	*0.600*	51.2	29.8	9.3	2.4
Tech	Did not know how to use their technology (software, computer, projector, etc.)	0.854	38.5	46.8	7.3	5.4	2.0	0.854	42.9	32.2	14.1	7.3

*A, appropriate knowledge; C, compatibility; D, discriminability; I, informative change; L, limited capacity; R, relevance; S, salience; Tech, problems with the technology*.
